# Identification of epigenetic factors regulating the mesenchyme to epithelium transition by RNA interference screening in breast cancer cells

**DOI:** 10.1186/s12885-016-2683-5

**Published:** 2016-08-31

**Authors:** Jean-Marc Gregoire, Laurence Fleury, Clara Salazar-Cardozo, Frédéric Alby, Véronique Masson, Paola Barbara Arimondo, Frédéric Ausseil

**Affiliations:** Unité de Service et de Recherche CNRS-Pierre Fabre n°3388 ETaC, CRDPF, 3 avenue H. Curien, BP 13652, 31035, Toulouse cedex 01, France

**Keywords:** Epithelium, Mesenchyme, Transition, RNAi, Screening, DOT1L, KAT5/Tip60

## Abstract

**Background:**

In breast cancer, the epithelial to mesenchyme transition (EMT) is associated to tumour dissemination, drug resistance and high relapse risks. It is partly controlled by epigenetic modifications such as histone acetylation and methylation. The identification of genes involved in these reversible modifications represents an interesting therapeutic strategy to fight metastatic disease by inducing mesenchymal cell differentiation to an epithelial phenotype.

**Methods:**

We designed a siRNA library based on chromatin modification-related to functional domains and screened it in the mesenchymal breast cancer cell line MDA-MB-231. The mesenchyme to epithelium transition (MET) activation was studied by following human E-CADHERIN (E-CAD) induction, a specific MET marker, and cell morphology. Candidate genes were validated by studying the expression of several differential marker genes and their impact on cell migration.

**Results:**

The screen led to the identification of 70 gene candidates among which some are described to be, directly or indirectly, involved in EMT like *ZEB1, G9a, SMAD5* and *SMARCD3*. We also identified the DOT1L as involved in EMT regulation in MDA-MB-231. Moreover, for the first time, *KAT5* gene was linked to the maintenance of the mesenchymal phenotype.

**Conclusions:**

A multi-parametric RNAi screening approach was developed to identify new EMT regulators such as KAT5 in the triple negative breast cancer cell line MDA-MB-231.

**Electronic supplementary material:**

The online version of this article (doi:10.1186/s12885-016-2683-5) contains supplementary material, which is available to authorized users.

## Background

In breast tumours, the epithelium to mesenchyme transition (EMT) is associated to early metastatic cell dissemination, drug resistance and high relapse risks [1]. During this epithelial cell dissemination, primary tumours acquire a mesenchymal phenotype [2]. Cytoskeletal rearrangements resulting in loss of cell polarity and morphology properties improve the migratory and invasive features of the cells [3]. Relapse risks are frequent for particularly aggressive cancer forms which display EMT and invasive properties often associated to CD44^high^ / CD24^-/low^ phenotype and present tumour initiating cell (TICs) features like auto-renewing and chemo-resistance [4–6]. Interestingly, the analysis of clinical samples indicates that metastases often closely look like the primary tumour in morphology and gene expression profile suggesting that the redifferentiation of the metastasizing cell may occur via a mesenchymal to epithelial transition (MET) [7]. Indeed, after MET, the cells look and expand to form a secondary tumour [8–10]. Strikingly, changes in cellular characteristics during a bona fide MET are to a large extent dependent on the upregulation of E-CAD and the repression of N-CADHERIN (N-CAD), both belonging to type-1 transmembrane proteins class regulated by the MET program [3]. As cell dissemination and tumour initiation are linked to MET in breast cancer, the identification of the targets involved in this biological pathway is critical for the discovery of novel therapies.

The role of epigenetic mechanisms in EMT of breast cancer cells is emerging [11]. Epigenetic is composed of chromatin modification (CM) such as DNA methylation, histone post-modifications that dictates access to DNA, thereby playing a major role in the regulation of transcription, DNA recombination, replication, and repair [12]. Higher-order chromatin structure is also an important regulator of gene expression during mammalian development, lineage specification [13] and shapes the mutational landscape of cancer [14]. Since chromatin modifications are reversible, epigenetic marks constitute ideal targets for therapeutic action.

Here, we aimed at identifying the regulators involved in MET as future therapeutic targets in breast cancer. MDA-MB-231 cell line was used as mesenchymal breast cancer model and RNA interference (RNAi) was used to identify the chromatin modifying domains involved in MET. RNAi-mediated gene silencing is a valuable tool widely used in drug discovery [15, 16] notably in high-throughput screening [17, 18]. A set of 729 chromatin modifying target genes were chosen according to the bioinformatic study of Pu et al. [19] and pools of four siRNA per target were designed.

Since E-CAD induction is a feature of MET, we followed the detection of E-CAD by fluorescence microscopy together with the change in cell morphology towards an epithelial phenotype. To confirm the siRNA hits, the expression of targeted genes and their impact on cell migration were measured. Thereby, the already described *G9a, SMAD5* and *SMARCD3* were identified to be involved in MET, as also *DOT1L* that has been recently published in this domain. Finally, for the first time, *KAT5* was found to be involved in MET.

## Methods

### Cell line and drug

MDA-MB-231 cells were grown in Dulbecco’s modified Eagle’s medium (DMEM-GlutaMAX^TM^-I from Gibco) supplemented with 10 % fetal bovine serum (Lonza). Cells were incubated at 37 °C with 5 % CO_2_ and subcultured twice weekly during the experimental period.

EPZ-5676 was purchased from ChemScene (USA). A DMSO stock solution (10 mM) was prepared and stored at −20 °C until ready for use. Working dilutions were prepared in DMEM just before use.

### SiRNA and miRNAs

The SMARTpool siRNA library (targeting 729 known and putative human chromatin modifiying genes) was purchased from Dharmacon (GE Healthcare) in ten 96-well plates (80 SMARTpool siRNAs/plate). The ON-TARGETplus siRNA SMARTpool against ZEB1 was purchased from Dharmacon (GE Healthcare) whereas the negative control siRNA (siScr) was purchased from Qiagen (AllStars Negative Control). The pre-miR-200a, pre-miR-200c and pre-miR Negative Control 2 were purchased from Ambion (Life Technologies) [20].

### siRNA screening and hits validation

MDA-MB-231 (3,000/well) were reverse transfected in 96-well plates, in duplicate, with SMARTpool siRNA library using Lipofectamine® RNAiMAX (Invitrogen) following the manufacturer’s instructions. The final concentration of each SMARTpool siRNA was 10nM in 100 μl medium per well. After 72 h, media were removed and cells were re-transfected (forward transfection) with SMARTpool siRNA at the same concentration as previously described. After 72 h, media were definitively removed and cells were washed one time with PBS1x before fixation with 3.7 % paraformaldehyde (Sigma-Aldrich) and permeabilization with 0.1 % Triton X-100 (Sigma-Aldrich). The plates were then blocked with PBS1x containing 2 % BSA plus 0.05 % Tween-20 (Sigma-Aldrich) overnight at 4 °C. Next, the plates were incubated with mouse anti-E-CAD antibody (1:200; BD Pharmingen) for 2 h at room temperature. After washing three times with PBS 1× plus 0,05 % Tween 20, the plates were incubated with a mixture of Alexa Fluor® 488 Donkey Anti-Mouse antibody (1:1000; Life Technologies), Texas-Red®-X Phalloidin (1:200; Life Technologies) and DAPI (1:2000; AAT Bioquest) for 1 h at room temperature, washed three times before analysis on the IN Cell Analyser 1000 (20×, GE Healthcare). Five fields per well were scanned and analysed. Each plate contained two positive controls (a SMART pool directed against *ZEB1* and a pre-miR200c) and two negative controls (cells treated with transfection reagent alone; and transfected with a scramble siRNA). For each transfection, the immunofluorescence of E-CAD was normalized to the cell number measured by DAPI staining. The data were normalized to the median signal of the plate and MAD (median absolute deviation) was used for hit selection [21]. For analysis, since the values measured for the ZEB1 positive control were between one or two MAD, hits were selected on this criteria: a MAD value superior to one. The MAD value was associated to cell morphological change analysis (Moreno-Bueno et al. [22]). For hit validation, E-CAD induction was measured by RT-qPCR and considered positive if two single siRNA out of the four of the pool were positive (Boutros et al. [23]). The significance of E-CAD induction was analysed using the Wilcoxon-Mann-Whitney test. A *p*-value <0.05 was considered statistically significant.

### RNA isolation

After two successive transfections, cells were harvested by trypsinization and total RNA was isolated using the RNeasy plus mini kit following the manufacturer’s instructions (Qiagen). The quantity and quality of the RNA were determined using the NanoDrop 2000 spectrophotometer (ThermoScientific).

### Quantitative RT-qPCR

cDNA was synthetized from 1 μg of total RNA using the SuperScript® VILO^TM^ cDNA Synthesis Kit according to the manufacturer’s instructions (Life Technologies). QRT-PCR was performed using SYBR® Green PCR Master Mix (Applied Biosystem) and a CFX384^TM^ Real-Time PCR Detection System (Bio-Rad). Gene expression was normalized to three endogenous control genes (hydroxymethylbilane synthase (*HMBS*), Peptidylprolyl Isomerase A (*PPIA*), Importin 8 (*IPO8*).

PCR primers were synthetized by Eurogentec. The following primer sequences were used.

For *DOT1L*, 5’-GCTGCCACCAGACTGACCA-3’(forward) and 5’-TCCTAGTTACCTCCAACTGTGCC-3’(reverse); for *KAT5* 5’-TCCCCAGGGGGAGATAATCGAG-3’(forward) and 5’-GCCAGGGGCCACTCATCTTC-3’ (reverse); for *E-cadherin* 5’-TCCCACCACGTACAAGGGTC-3’(forward) and 5’-GGGGGCATCAGCATCAGTCA-3’(reverse); for *CD24* 5’-AACTAATGCCACCACCAAGG-3’(forward) and 5’-GACGTTTCTTGGCCTGAGTC-3’(reverse); for *TSPAN13* QuantiTect Primer Assay (Qiagen); for *HMBS* 5’-ATACAGACGGACAGTGTGGTGGC-3’(forward) and 5’-CCCTGTGGTGGACATAGCAATGA-3’(reverse); for *PPIA* 5’-GAGCACTGGAGAGAAAGGATTTGGTT-3’(forward) and 5’-CGTGTGAAGT CACCACCCTGACA-3’(reverse); for *IPO8* 5’-GAGTGTGAGGGTCAAGGGGATG-3’(forward) and 5’-AAAGTGCTGCCTAATGCCAGATG-3’(reverse).

### Migration assays

Migration assays were performed with the Oris^TM^ Cell Migration Assay following the manufacturer’s instructions (PLATYPUS Technologies). Briefly, after two successive transfections, cells were harvested by trypsinization and counted. For each transfection, 80.000 cells/well were seeded and allowed to adhere for 24 h. Stoppers were removed and the plate was incubated to permit cell migration for 24 h. The cells were labelled with calcein AM (Life Technologies) and the fluorescence was detected using a Typhoon Trio (GE-Healthcare). The effects on cell migration were estimated by cell surface area calculation using Image J program (National Institutes of Health Image). Each experiment was done in triplicate with two independent repeats.

## Results and discussion

### Design of the 729 siRNA pool library

The siRNA pool library is directed against 729 known or predicted chromatin modifier genes like chromatin-remodeling factors (KATs, HDACs, KMTs and KDMs), transcriptional coactivators or corepressors (Additional file 1). Substantial evidences show that the chromatin modifying factors exhibits distinct protein domains that perform specific functions, such as SET domain (a catalytic domain of many histone lysine methyl-transferases), Bromodomain (responsible for recognition of acetylated histone lysine) or Chromodomain (responsible for binding of methylated histone lysine) [24–26]. The library, which includes four independent siRNAs for each targeted gene, was designed according to an orthology-based computation analysis of the Pfam protein database looking for the protein domains involved in chromatin modification [19, 24–27]. In this study, the authors predicted 397 novels CM genes (coding for 329 proteins) in humans in addition to 398 experimentally verified ones to propose a library of genes in chromatin modification. Here, the siRNA library was generated by deleting unvalidated gene sequences and adding genes involved in DNA methylation to obtain the 729 siRNA pools library (Additional file 1 for the list of the RNAi bank).

### Screening strategy’s steps

**Fig. 1:**
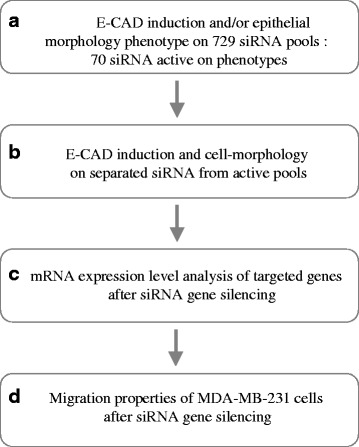
Screening strategy. A four step process was used to identify MET inducer gene candidates. **a** The primary screening was performed on 729 siRNA pools targeting 729 genes selected for chromatin structure maintenance. From E-CAD induction measurements and cell morphology observations, 70 pools were identified. **b** Deconvolution analysis: E-CAD induction and cell morphology were analysed for each siRNA contained in the active pools. **c** Transcript quantification was done by RT-qPCR to control gene knockdown. **d** Cell migration study: the lost of the mesenchyme phenotype was associated to impaired migration capabilities for several siRNA

To identify new chromatin modifying genes involved in the maintenance of the mesenchymal state, a four step strategy was performed (Fig. 1). The triple-negative breast cancer (TNBC) cell line model MDA-MB-231 was chosen because it’s representative of the mesenchymal-like phenotype of cancer cells and represents one of the most aggressive human cancer cells when grafted in mice [28, 29]. Interestingly, HDACi inhibition initiates a partial MET which is associated to decreased tumorigenesis in vivo [30] indicating that by acting on the epigenetic regulation it is possible to reverse the mesenchymal phenotype. In addition, this cell line has a relatively high percentage of CD44^+^/CD24^-/low^ cells which have been reported to have stem/progenitor cells properties [4] and enhanced invasive properties [31].

The MDA-MB-231 cells do not express, or weakly, E-CAD which is silenced by methylation of its promoter [28]. The MET is partly characterized by the reactivation of E-CAD a marker of the epithelial state. Thus, the first step of the strategy consisted in screening the 729 siRNA pools on these cells to identify the pools of siRNA that induced E-CAD as followed by immunofluorescence. In parallel, epithelial cell morphology was followed by F-ACTIN immunofluorescence staining. Second, the 4 siRNA of each active pool were tested separately on both E-CAD induction and cell morphology. Third, the down-regulation of the targeted genes was confirmed by RT-qPCR. Fourth, the effect of the siRNA was further validated by inhibition of the migration properties of the cells.

### Cell-based assay validation

The microRNA-200 (miR-200) family has emerged recently as important regulators of EMT/MET [32]. This family comprises five members expressed from two distinct polycistronic transcripts (miR-200b ~ 200a ~ 429 and miR-200c ~ 141) and, on the basis of their ‘seed’ sequence [33], can be separated in two functional groups (miR-200b/200c/429 and miR-141/200a). The miR-200c is known to be involved in cells undergoing EMT/MET [20, 34].

**Fig. 2:**
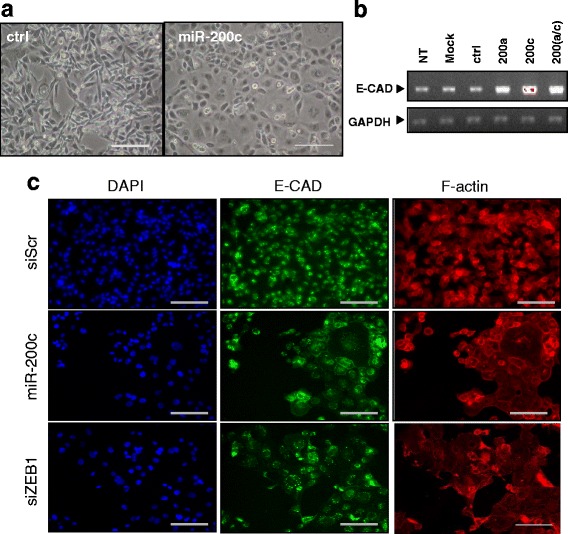
Cell based assay development and validation. **a** MDA-MB231 cells were transfected with a pre-miRNA negative control (ctrl; 5nM) or pre-miR-200c (5nM). Phase contrast images were taken at 6 days after 2 successive transfections (magnification, ×10). **b** MDA-MB-231 cells were transfected as in A with a pre-miR negative control scramble (ctrl), pre-miR-200a (200a), pre-miR-200c, mix of pre-miR200a + pre-miR-200c (200a/c) (10 nM) and after 6 days the expression of E-CAD and GAPDH were studied by RT-qPCR. Non treated cells (NT); Mock-transfected cells (Mock). **c** Immunofluorescence staining of E-CAD (*green*) and texas-red phalloidin staining of F-ACTIN (*red*) in cells transfected as in (**a**) with scramble (siScr), pre-miR-200c and a ZEB1-specific siRNA pool (10 nM). Cells are counterstained with DAPI (*blue*) to visualize nuclei. Scale bars, 100 μm

The miR-200c and a miRNA negative control were used as positive and negative controls respectively. The comparison of miR-200c and miRNA negative control transfected cells in phase contrast microscopy showed a dramatic change of cell morphology, from an elongated fibroblast-like shape with pronounced cellular scattering to a cobblestone-like epithelial phenotype (Fig. 2a). RT-qPCR analysis revealed a significant increase in the expression of the epithelial marker E-CAD mRNA in miR200 family (miR-200b and miR200a) transfected cells (Fig. 2b). The immunofluorescence analysis of E-CAD reinforced this result. In several cancer cell types, the miR-200 family is able to enforce an epithelial state by inhibiting the E-CAD transcriptional repressor ZEB1 [33, 35]. In our model, cells transfected with miR-200c, or a specific SMARTpool directed against ZEB1, showed a strong E-CAD cellular membrane staining and a discrete nuclear staining whereas MDA-MB-231 cells transfected with a miRNA negative control (data not shown) or an irrelevant siRNA only showed a weak nuclear staining (Fig. 2c). As E-CAD nuclear staining was unexpected, we conducted the same experiment with a second antibody directed against E-CAD obtaining the same result (data not shown). Finally, we observed an increase in E-CAD signal and F-ACTIN staining with phalloidin clearly revealed the cuboidal phenotype, typical of epithelial cells, of miR-200c and siZEB1 transfected cells (Fig. 2c). Taken together, these experiments validate miR-200c and siZEB1 as inducers of MET in MDA-MB-231 cells.

### siRNA screening reveals genes potentially involved in MET

Transfection reagent, cell number and siRNA concentrations were optimized to obtain a maximum of 20 % reduction in cells viability when transfected with the irrelevant (non silencing) siRNA compared to mock-transfected (cells treated with transfection reagent, no siRNA) and untransfected cells. Screening conditions were also optimized to ensure high transfection efficiency by using a siRNA pool targeting the essential gene, *KIF11* (*EG5*) (data not shown).

To calculate E-CAD induction in the screen, a statistical method based on MAD calculation was used [21]. This method enabled a significant E-CAD induction detection of miR-200c and siZEB1 transfected cells. The MAD calculation method identified two groups of hit SMART pools. Group A contains 53 genes whose individual knockdown induced a statistically significant increase in E-CAD cellular fluorescence (threshold ≥ one MAD) and morphological changes associated to a partial reversal of the mesenchymal phenotype and group B targeting 17 genes, which knockdown induced only morphological changes. Due to cell and siRNA transfection heterogeneity, we also considered these genes because they might be associated with modifications of adhesion properties and linked to metastatic process.

### Hit validation

**Fig. 3:**
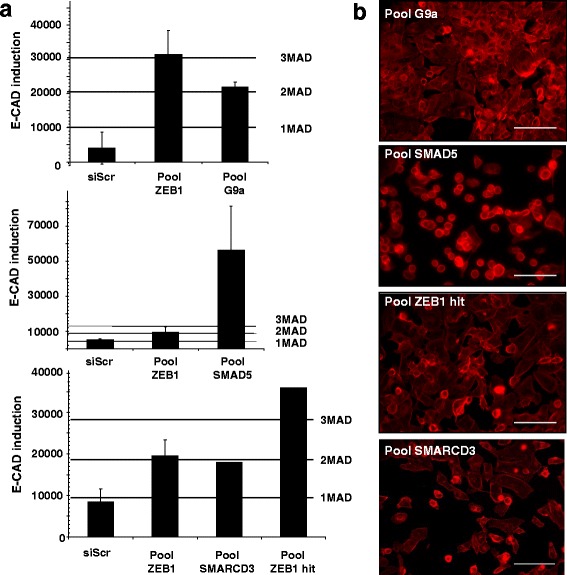
High throughput functional screen to detect genes potentially involved in MET. **a** Four examples obtained in the initial screen. E-CAD expression was normalized to cell number then data were normalized to the median of SMARTpools in the same plate (*n* = 80 SMARTpools/plate). Threshold and hits selection were based on MAD calculation. **b** Morphological changes are revealed by F-ACTIN staining (*red*) as described before. Scale bars, 100 μm

**Fig. 4:**
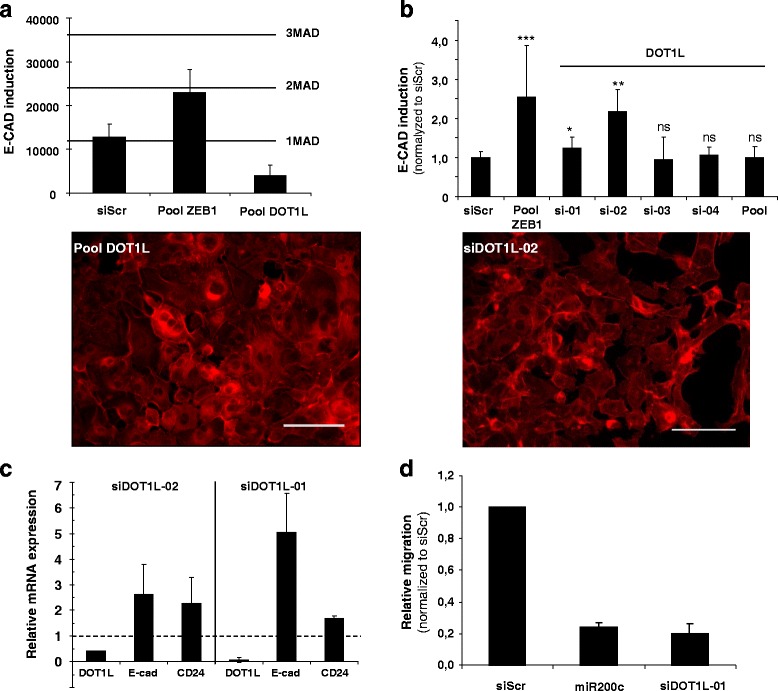
DOT1L silencing induces MET and CD24 mRNA expression in MDA-MB-231 in vitro. **a** Quantitative and qualitative analysis after transfection with DOT1L SMARTpool. **b** Quantitative and qualitative analysis after transfection with four individual siRNA and the DOT1L SMARTpool in MDA-MB-231 cells. Individual siRNAs significantly induced E-CAD (siRNA 1, 2) and morphological changes (siRNA 2) compared with siScr transfected cells. Data are presented as the mean ± sd of 2 independent experiments each with 3 biological replicates (* *p* < 0.05, **p < 0.01, ***p < 0.001 compared with siScr determined by Wilcoxon-Mann Whitney’s test). **c** RT-qPCR quantification of *DOT1L*, *E-CAD* and *CD24* transcripts using specific primers in MDA-MB-231 cells transfected with individual siRNA 1 (10 nM) and 2 (0.1 nM). The columns represent the mean expression ± sd of 2 independent experiments normalized to siScr. **d** Individual siRNA 1 (10 nM) reduced the migration compared with siScr or miR-200c transfected cells. Data are presented as the mean ± sd of 2 independent experiments each with 2 biological replicates. Scale bars, 100 μm

**Fig. 5:**
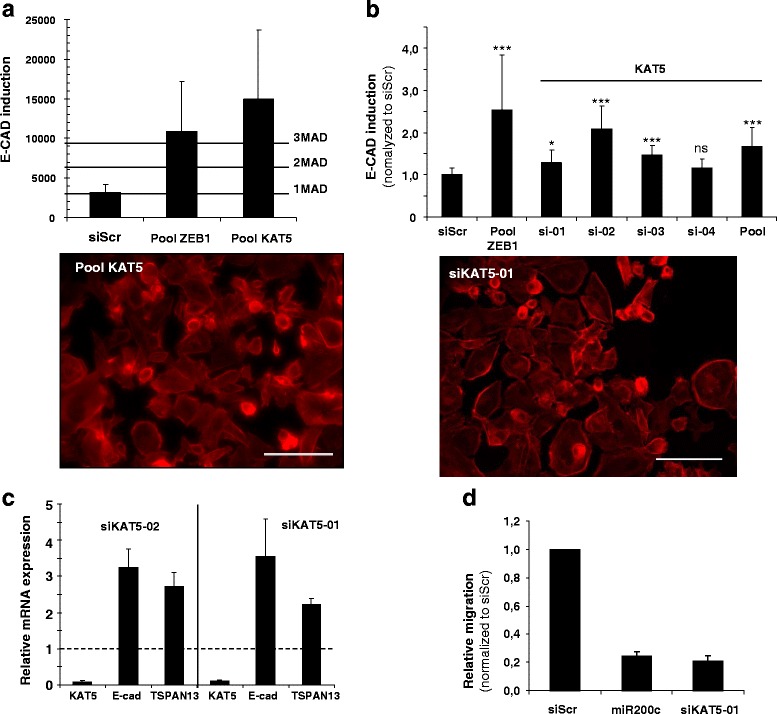
KAT5 silencing induces MET and TSPAN13 mRNA expression in MDA-MB-231 in vitro. **a** Quantitative and qualitative analysis after transfection with KAT5 SMARTpool of E-CAD induction and cell morphology. **b** Quantitative and qualitative analysis after transfection with four individual siRNA and the KAT5 SMARTpool in MDA-MB-231 cells. Data are presented as the mean ± sd of 2 independent experiments each with 3 biological replicates (* *p* < 0.05, **p < 0.01, ***p < 0.001 compared with siScr determined by Wilcoxon-Mann Whitney’s test). **c** RT-qPCR quantification of *KAT5, E-CAD* and *TSPAN13* transcripts using specific primers in MDA-MB231 cells transfected with individual siRNA 1 (10nM) and 2 (1nM). The columns represent the mean expression ± sd of 2 independent experiments normalized to siScr. **d** Individual siRNA 1 (10nM) reduced the migration compared with siScr or miR-200c transfected cells. Data are presented as the mean ± sd of 2 independent experiments each with 2 biological replicates. Scale bars, 100 μm

The fact that several target genes were already known to be involved, directly or indirectly, in MET conforted our strategy. These genes include in particular *G9a* [36], *SMARCD3* [37], *SMAD5* [38] and *ZEB1*, which is also the positive control (Fig. 3) [39]. We then focused on two genes: DOT1L (group B) and KAT5 (Tip60) (group A) (Fig. 4a and 5a). DOT1L is a histone H3 lysine 79 methyltransferase whose inhibition increases the yield of induced pluripotent stem cells (iPSCs) [40]. It was described very recently as an EMT modulator through a bioinformatic analysis of a large breast cancer genetic database [41]. KAT5 is an histone acetyltransferase (HAT) required to maintain characteristic features of ESCs [42]. It is linked for the first time here to the MET regulation.

To confirm the initial results and eliminate false positives due to off-target effects, we repeated the primary screen using deconvoluted single siRNAs targeting *DOT1L* and *KAT5* (Figs. 4b and 5b). For each target, two out of four siRNAs tested present in the pools reproduced the observed primary screen phenotypes. Most remarkably, two out of four siRNAs targeting *DOT1L* were found to be significant E-CAD inducers when tested individually placing the *DOT1L* also in group A (Fig. 4b). The difference between the SMART pool and the single siRNA could be due to the siRNA potency. The effect of the *DOT1L* and *KAT5* knockdown was further demonstrated by RT-qPCR and correlated to an increase in E-CAD mRNA and to a decrease in DOT1L or KAT5 mRNA levels. The implication of the two genes in MET regulation and stem/progenitor cell phenotypes was investigated by following the expression of mesenchymal and epithelial marker genes such as vimentin, *ZEB1*, E-cadherin, Tetraspanin 13 (*TSPAN13*), Occludin (*OCLN*) and the stem/progenitor cell surface markers CD24 and CD44. Among the seven markers studied, changes in *E-CAD* and *CD24* expression were observed in response to *DOT1L* silencing and in *E-CAD* and *TSPAN13*, a potent breast cancer suppressor gene [43], after *KAT5* knockdown (Fig. 5c). The different marker expression profiles observed after *DOT1L* or *KAT5* silencing may reflect partial MET [44].

A functional change associated with EMT is an increase in migration and/or invasion capacities [45]. As *DOT1L* or *KAT5* silencing strongly decreases migration of MDA-MB-231, in vitro*,* after two successive transfections with no major effect on cell viability (Fig. 5d), we argued that DOT1L and KAT5 were involved in different steps of MDA-MB-231 differentiation and could be potential therapeutic targets to inhibit TNBC metastasis.

**Fig. 6:**
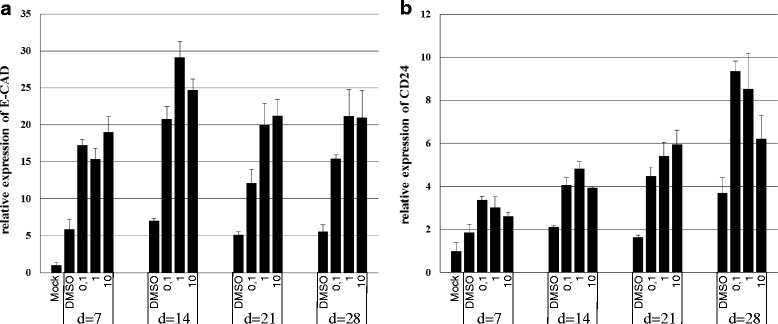
Pharmacological inhibition of DOT1L induces E-cadherin and CD24 expression in MDA-MB-231 in vitro. Cells were treated with dose effects of EPZ-5676 (0.1 μM, 1 μM, 10 μM) or 0.1 % DMSO for the indicated days (noted d), followed by mRNA extraction and RT-qPCR with specific primers. **a** E-CAD expression relative to non treated cells. **b** CD24 expression relative to non treated cells. Data in **a** and **b** are the mean of RT-qPCR replicates from a representative experiment, and error bars indicate SEM. The experiments were run 2 times

Finally, to confirm DOT1L as therapeutic target, we treated MDA-MB-231 cells with a potent and selective DOT1L inhibitor EPZ-5676 [46]. After a 7 days treatment, this drug showed a strong dose-dependent increase in E-CAD mRNA and a slight upregulation of CD24 mRNA (Fig. 6). These results were totally consistent with gene expression changes observed after *DOT1L* silencing and confirmed the role of DOT1L in MDA-MB-231 CSC-like cells differentiation.

## Conclusions

From this RNAi-based phenotypic screening, we have identified a set of 70 potentials hits, that may promote the conversion of the highly invasive mesenchymal-like cells MDA-MB-231 into a more differentiated and less aggressive phenotype. *KAT5* and *DOT1L* gene downregulation induced E-CAD expression and epithelial morphological changes. The process was validated by the finding of hits such as *ZEB1, G9a, SMAD5, SMARCD3,* already reported in the literature to be implicated in the regulation of EMT/MET. Indeed, ZEB1 is a well known transcriptional repressor directly implicated in the control of EMT [34] that we used as positive control to design the screening assay. The knock-down of G9a, a histone methyltransferase, restored E-CAD expression, caused morphological changes and attenuated migratory and invasive capacity of MDA-MB-231 cell line in vitro and in vivo [36]. Furthermore, SMAD5 phosphorylation induced by an aberrant Aurora-A kinase activity, led to its nuclear activation and ultimately contributed to the development of EMT, stemness and tumor progression in human breast cancer cell line MCF-7 [38]. Finally, the silencing of SMARCD3/Baf60c, a SWI/SNF chromatin-remodeling factor, gives a strong MET by Wnt5a downregulation in EpCAM^-^ SUM149 or SUM229 subpopulation [37].

Among the 70 gene candidate as putative MET regulators, DOT1L and KAT5 were found to both induce E-CAD and to promote an epithelial morphological phenotype in MDA-MB-231. DOT1L was previously identified as a modulator of pluripotent stem cells (iPSCs) reprogramming [40] and shown to methylated the H3K79 mark which is critical in Mixed Lineage leukemia (MLL) by enhancing expression of leukemogenic genes like *HOXA9* and *MEIS1* [47]. In vivo, administration of a DOT1L selective inhibitor increased the lifespan of mice grafted with a preclinical model of MLL [46]. In colon cancer, DOT1L increases cancer stemness and tumorigenic potential by inducing the core stem cell genes *NANOG*, *SOX2* and *Pou5F1* [48]. In this study, DOT1L silencing and chemical inhibition by EPZ5676 induced E-CAD and CD24 expression and reduced the migration properties of MDA-MB-231 cells. These results support the idea that DOT1L is involved in EMT and in the maintenance of CD44^+^/CD24^-^ cancer stem cells present in MDA-MB-231 cell line. These results are in agreement with those published by Zhang et al. in 2014, showing that DOT1L was a potential drug target for breast cancer and metastatic disease [41].

Finally, this siRNA screening led to the identification of KAT5, a target never described in MET regulation up today. KAT5 is a HAT with regulatory functions in signalling, transcriptional activation, DNA repair, apoptosis and cell cycle progression [49]. In embryonic stem cells (ESCs), one of the most important functions of KAT5 is to repress developmental genes [42]. In basal-like breast cancer, the TWIST protein, a well known EMT inducer [50], is specifically diacetylated by KAT5 to interact with BRD4 and activate WNT5A. As a result of this interaction, it induces invasion and increases (CSC)-like properties and tumorigenicity. Lastly, in radioresistant subpopulations of breast cancer cells induced by irradiation, ATM, a protein activated by KAT5 acetylation, is hyperactivated and mediates stabilization of ZEB1, another well known EMT inducer, in breast cancer and other types of solid tumours [51, 52]. Altogether, combined with the fact that *KAT5* silencing induces E-CAD and TSPAN13 expression, it strongly suggests that a KAT5 inhibitor can induce TNBC differentiation (basal-like subtype) and, in combination with classical chemotherapeutic agents, reduces the number of metastases [53]. Another study shows a metastatic suppression function of KAT5 in a prostate cancer model highlighting the fact that EMT regulation is strongly tissue dependant [54]. Moreover, as a result of the direct relationship between KAT5 and ATM kinase, our findings may highlight the critical role of the DNA damage response (DDR) in tumorigenesis and metastasis in the basal subtype of breast cancer [55, 56].

In conclusion, the screening method we developed enables the identification of validated and putative targets involved in the mesenchyme phenotype maintenance of triple negative breast cancer cells. These targets need to be further investigated to demonstrate their antitumoral effect in animal models and patients.

## Additional file

Additional file 1: Raw Data. An excel file containing 10 spreadsheets. Each spreadsheet has the name of the figure in which the data were used. (XLS 788 kb)

## Abbreviations

CM: Chromatin modification
DDR: DNA damage response
E-CAD: E-CADHERIN
EMT: Epithelial to mesenchyme transition
ESCs: Embryonic stem cells
HMBS: Hydroxymethylbilane synthase
IPO8: Importin 8
iPSCs: Induced pluripotent stem cells
MAD: Median absolute deviation
MET: Mesenchyme to epithelium transition
N-CAD: N-CADHERIN
OCLN: Occludin
PPIA: Peptidylprolyl isomerase A
RNAi: RNA interference
TICs: Tumour initiating cells
TNBC: Triple-negative breast cancer
TSPAN13: Tetraspanin 13
